# The Impact of Frailty on the Risk of Conversion from Mild Cognitive Impairment to Alzheimer’s Disease: Evidences from a 5-Year Observational Study

**DOI:** 10.3389/fmed.2017.00178

**Published:** 2017-10-23

**Authors:** Alessandro Trebbastoni, Marco Canevelli, Fabrizia D’Antonio, Letizia Imbriano, Livia Podda, Lidia Rendace, Alessandra Campanelli, Valentina Celano, Giuseppe Bruno, Carlo de Lena

**Affiliations:** ^1^Department of Neurology and Psychiatry, Sapienza University of Rome, Rome, Italy

**Keywords:** mild cognitive impairment, Alzheimer’s disease, dementia, frailty, aging

## Abstract

The frailty construct has increasingly been adopted in the field of cognitive disorders. The aim of the present study was to measure frailty in a cohort of individuals with mild cognitive impairment (MCI) and to explore whether frailty measures may consent to predict the risk of conversion to dementia. We retrospectively reviewed the clinical charts of outpatients with amnesic MCI (aMCI) consecutively recruited at our Department, and followed-up for 5 years. Individual frailty status was measured by means of a frailty index (FI) consisting of 39 deficits (including signs, symptoms, diagnoses, and disabilities). Univariate analyses were used to compare the socio-demographic and clinical characteristics between subjects converting or not converting to probable Alzheimer’s disease (AD) dementia over the follow-up. Risk for conversion to AD dementia was assessed using Cox regression models. Ninety-one subjects with aMCI (mean age 72.7, SD 7.1 years; women 49.5%) were consecutively recruited over a period of 12 months. Low levels of frailty were documented in the sample (mean FI score 10.0, SD 5.3). A statistically significant correlation between age and FI was observed. Overall, 58 participants converted to AD dementia over time. The Cox regression analysis showed that age (HR: 1.04, 95% CI: 1.00–1.08), male sex (HR: 0.52, 95% CI: 0.30–0.91), Mini–Mental State Examination score (HR: 0.85, 95% CI: 0.77–0.94), and FI (HR: 1.11, 95% CI: 1.05–1.18) were all significantly associated with the probability of MCI conversion. Individual’s frailty status may increase the risk of conversion from a condition of MCI to overt AD dementia. The adoption of constructs comprehensively reflecting the biological decline of the aging subject may add useful estimates and information in the clinical approach to cognitive disorders.

## Introduction

Frailty has been conceptualized as “a medical syndrome with multiple causes and contributors that is characterized by diminished strength, endurance, and reduced physiologic function that increases an individual’s vulnerability for developing increased dependency and/or death” (1). This construct has increasingly been adopted in order to capture the biological decline of the aging individual and his/her risk profile for negative health-related outcomes (2). Moreover, it is growingly acquiring public health relevance as it may support the realignment of models of care to the changing needs of our aging populations (3).

In these last years, the relationship between frailty and cognition has triggered special interest. The contribution of cognitive skills and capacities to the individual’s vulnerability and resiliency has more consistently been considered and recognized (4). Cross-sectional analyses have repeatedly shown that frail individuals have lower cognitive performance compared with non-frail persons (5, 6). Accordingly, several longitudinal studies have documented a higher risk of incident cognitive impairment and dementia among frail subjects (5). More recently, frailty indexes (FIs) have been found to predict poorer outcomes (i.e., mortality, institutionalization, faster cognitive worsening) in populations of patients already exhibiting overt dementing conditions (7, 8). However, to our knowledge, no study has yet explored the impact of the individual frailty status on the clinical trajectories over time of subjects with milder cognitive deficits.

The aim of the present study was to measure frailty in a cohort of individuals with mild cognitive impairment (MCI) and to explore whether frailty measures may consent to predict the risk of conversion to dementia.

## Materials and Methods

### Setting and Participants

The present study was conducted at the Department of Neurology and Psychiatry of the “Sapienza” University of Rome (Italy). We retrospectively reviewed the clinical charts of outpatients with amnesic MCI (aMCI) consecutively recruited at our Department between April 2011 and April 2012 and followed up with clinical and neuropsychological evaluations (at least twice a year) for 5 years.

Amnesic MCI was defined according to the International Working Group criteria (9). To be included, subjects should have: (1) a self-reported cognitive concern confirmed by the caregiver; (2) the evidence of a lower performance in the memory domain or in the memory and other cognitive domains; (3) the complete preservation of independence in functional abilities; and (4) at least two clinical and neuropsychological assessments per year over an observation period of 5 years. Probable Alzheimer’s disease (AD) dementia was diagnosed according to the National Institute on Aging-Alzheimer’s Association criteria (10). A comprehensive neuropsychological assessment was performed in order to define aMCI and dementia and to evaluate cognitive changes over time. The test battery included the following standardized tests: Rey Auditory Verbal Learning Test (11, 12), Babcock Story Recall Test (13), Corsi Block-Tapping Test (13, 14), Digit Span Test (14), Visual Search Matrix Test (13), Boston Naming Test (11, 15), Verbal Semantic Fluency Test (11, 13), Verbal Phonemic Fluency Tests (11), Clock Drawing Test (16), Frontal Assessment Battery (17), Mini–Mental State Examination (MMSE) (18), and Clinical Dementia Rating Scale (19).

The cohort was divided in two groups of subjects based on the outcome of cognitive disturbances at the end of the observation period: (1) “MCI converters”: exhibiting a clinical progression toward a probable AD dementia and (2) “MCI non-converters”: whose cognitive and functional abilities either remained stable or improved during the follow-up.

Patients and caregivers (or legal guardians when necessary) provided written informed consent for allowing the utilization of the collected data for research purposes (as required by the local Ethics Committee). Data used in the present analyses were retrieved from medical charts where information was recorded as part of the standard clinical routine. In particular, comorbidities were defined on the basis of: (a) self-reports concerning previous diagnoses and/or laboratory findings and/or (b) available medical documents and/or (c) available medical prescriptions.

### Socio-Demographic and Clinical Variables

Socio-demographic (i.e., age, sex, and education) and clinical (i.e., comorbidities, physical and neurological examination, concomitant therapies, duration of cognitive symptoms) data were abstracted by the clinical charts of participants. Measures of global cognitive performance, assessed through the MMSE were also collected.

### Frailty Assessment

Frailty was measured by means of a FI, generated following a standard procedure (20) by computing 39 age-related, multidimensional deficits (including signs, symptoms, diagnoses, and disabilities) retrospectively resumed by the clinical charts (Table 1). Each item included in the FI was coded so that a value of 0 indicated the absence of the deficit and a value of 1 its presence. The FI was calculated as the ratio between the number of deficits presented by the individual and the number of considered deficits (i.e., 39) multiplied per 100 (in order to better show its statistical properties). Thus, the FI potentially ranged between 0 (no deficit) and 100 (all deficits).

**Table 1:** Items included in the computation of the 39-item frailty index.

1.	Hypertension
2.	Dyslipidemia
3.	Diabetes
4.	History of TIA
5.	History of stroke
6.	Ischemic heart disease
7.	Arrhythmia
8.	Chronic heart failure
9.	Gastric disorder
10.	Intestinal disorder
11.	Thyroid disease
12.	Cancer
13.	Arthritis
14.	Osteoporosis
15.	COPD
16.	Renal failure
17.	Cirrhosis
18.	Hematologic disease
19.	Peripheral artery disease
20.	Hearing impairment
21.	Vision impairment
22.	Parkinsonism
23.	Focal neurological signs
24.	Peripheral neuropathy
25.	Vascular encephalopathy (neuroimaging)
26.	Obesity (BMI ≥ 30)
27.	Underweight (BMI < 18.5)
28.	Depression
29.	Anxiety
30.	Sleep disorders
31.	Irritability
32.	Language disturbances
33.	Spatiotemporal disorientation
34.	Dizziness
35.	Falls
36.	Balance disorder
37.	Involuntary weight loss (≥4.5 kg in the last 6 months)
38.	Urinary incontinence
39.	Mobility disability (inability to walk 400 m)

*BMI, body max index; COPD, chronic obstructive pulmonary disease; TIA, transient ischemic attack*.

### Statistical Analysis

Statistical analyses were performed using the Statistical Package for Social Science for Mac (version 21, IBM Corporation, New York, NY, USA). Univariate analyses were conducted to compare the baseline data between “MCI converters” and “MCI non-converters.” Cox regression models were performed to measure the associations between the variables identified as significant or at borderline level of statistic significance in the univariate analyses and time to develop AD dementia, controlling for sex and age of participants. Hazard ratios with relative 95% confidence intervals were estimated. Sensitivity analyses stratified for MMSE scores were also conducted. Spearman’s correlations were used to assess the strength and direction of the relationship between age and FI. Statistic level of significance was set at *p* < 0.05.

## Results

**Figure 1:**
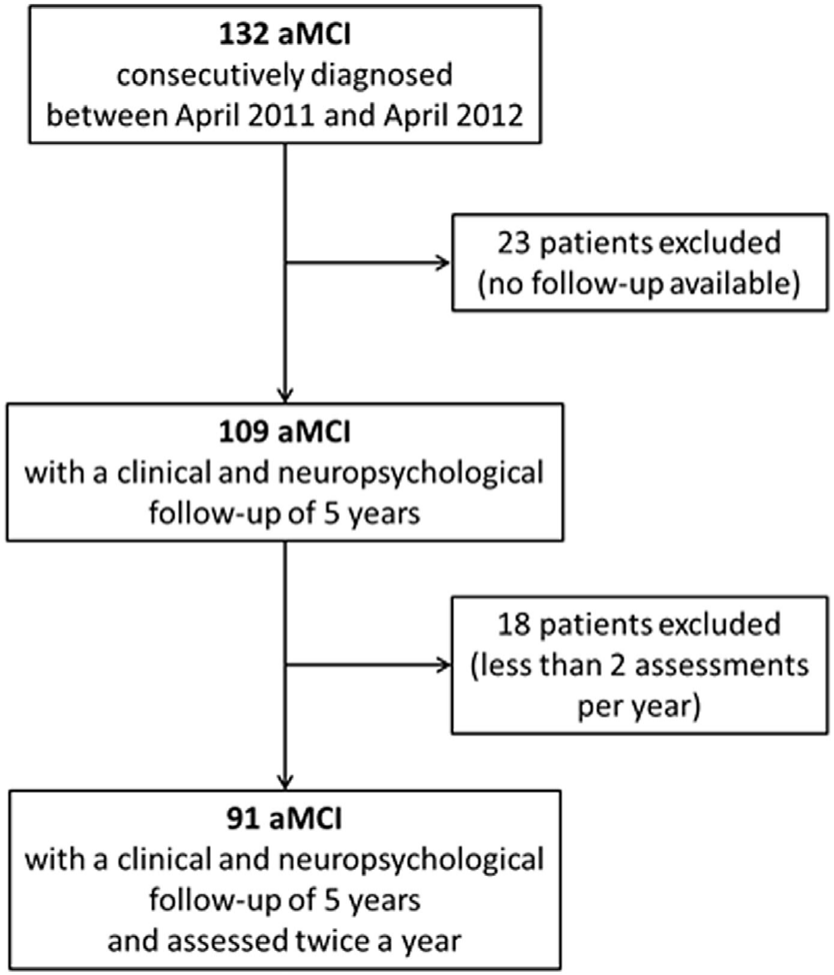
Flow chart of the study. One hundred thirty-two amnesic MCI (aMCI) patients were initially enrolled. Forty-one participants were retrospectively excluded (23 resulted were lost to follow-up; 18 did not undergo two or more clinical and neuropsychological assessments per year). Data from 91 aMCI subjects were finally considered for the present analyses.

**Figure 2:**
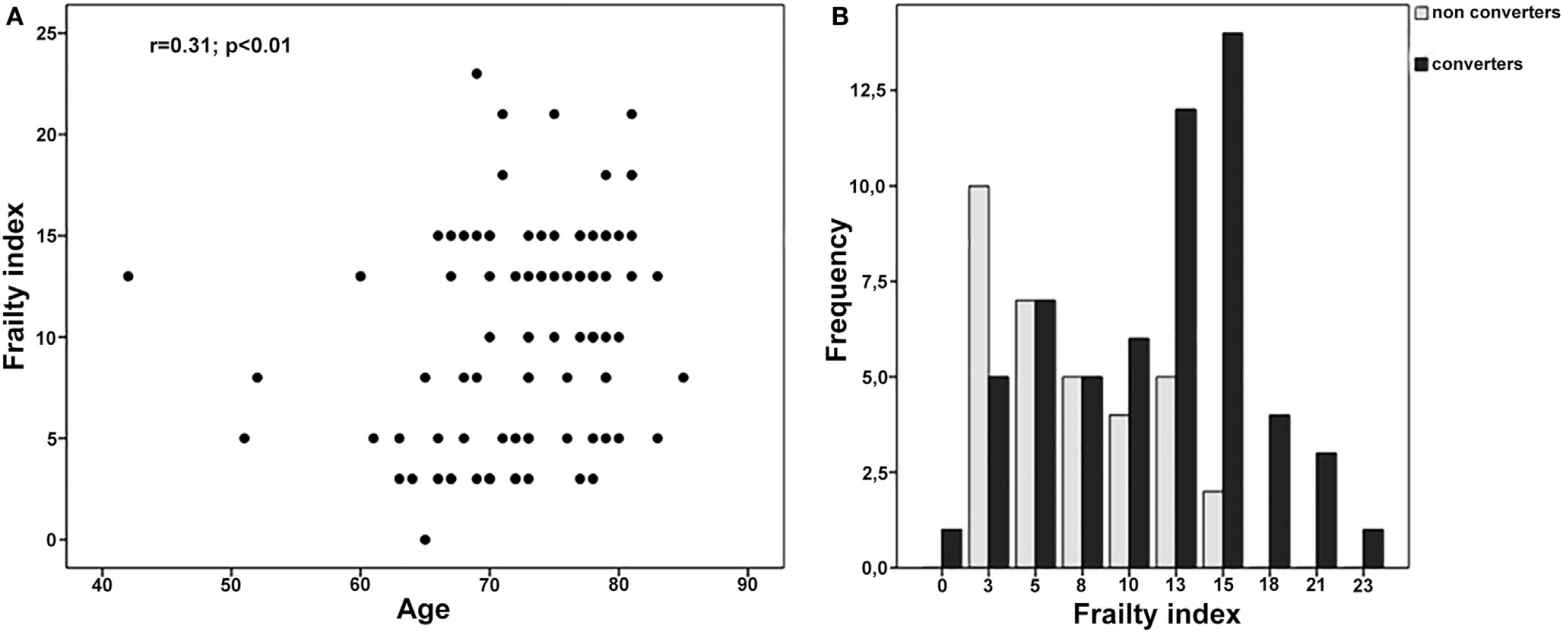
**(A)** Correlation between age and frailty index (FI) in the overall sample. **(B)** FI values among MCI converters and MCI non-converters.

One hundred thirty-two subjects were consecutively diagnosed with aMCI between April 2011 and April 2012 at our Department. The retrospective analysis showed that 109 of them were followed-up for the next 5 years. Nevertheless, only 91 subjects (women 49.5%) received two or more clinical and neuropsychological evaluations per year and were, thus, finally considered for the present analyses (Figure 1; Table 2). Participants had a mean age of 72.7 (SD 7.1) years and a mean educational level of 7.7 (SD 3.6) years. MMSE values at the baseline (mean 25.4, SD 2.8) indicated a globally preserved cognitive functioning. Low levels of frailty were documented in the sample (mean FI score 10.0, SD 5.3). Accordingly, none of the subjects resulted as frail [i.e., FI score ≥ 25.0 (8)]. A statistically significant correlation between age and FI was observed (Spearman’s *r* = 0.31; *p* < 0.01) (Figure 2A).

**Table 2:** Baseline sociodemographic and clinical characteristics of the sample according to MCI outcomes.

	Mild cognitive impairment (MCI) converters (*n* = 58)	MCI non-converters (*n* = 33)	*p*-Value
Age (years)	74.4 ± 4.9	69.7 ± 9.2	<0.01
Sex (women)	56.9	36.4	0.08
Education time (years)	7.3 ± 3.6	8.5 ± 3.4	0.13

MCI subtype			0.71
Single-domain aMCI	41.4	45.5	
Multiple-domain aMCI	58.6	54.5	

Hypertension	50.0	39.4	0.33
Dyslipidemia	34.5	33.3	0.91
Diabetes	12.1	0.0	0.04
Ischemic heart disease	13.8	9.1	0.51
Stroke	0.0	3.0	0.18
TIA	3.4	3.0	0.91
Chronic renal failure	0.0	3.0	0.18
COPD	0.0	3.0	0.18
Depression	41.4	42.4	0.92
Anxiety	24.1	18.2	0.51
Duration of cognitive disturbances (months)	25.8 ± 13.0	21.6 ± 11.6	0.12

MMSE	24.7 ± 3.0	26.7 ± 1.9	≤0.001
Frailty index	11.6 ± 5.3	7.3 ± 4.1	≤0.001

*Data are expressed as % or mean ± SD*.

*aMCI, amnesic mild cognitive impairment; COPD, chronic obstructive pulmonary disease; MMSE, Mini–Mental State Examination; TIA, transient ischemic attack*.

Over a follow-up of 5 years, 58 subjects converted from MCI to probable AD dementia, whereas 33 did not exhibit a clinical worsening. At the basal evaluation, “MCI converters” were older, more severely cognitive impaired, and exhibited a higher prevalence of diabetes compared to “MCI non-converters” (Table 2). Moreover, subjects converting to dementia had significantly higher mean scores at the FI (11.6, SD 5.3 vs. 7.3, SD 4.1; *p* < 0.001), indicating greater levels of frailty (Table 2 and Figure 2B).

The Cox regression model, adjusted for age and sex, showed that increasing age, male sex, lower MMSE scores, and higher FI scores were all significantly associated with an increased probability of MCI conversion (Table 3). The positive association between FI and the risk of conversion was also confirmed when restricting the analyses to only those subjects exhibiting normal MMSE scores (i.e., ≥24) (HR: 1.20; 95% CI: 1.05–1.19; *p* < 0.001) or the highest level of cognitive performance (i.e., MMSE ≥ 27, upper quartile of the distribution) (HR: 1.20; 95% CI: 1.05–1.19; *p* < 0.001).

**Table 3:** Cox regression analysis of factors predicting MCI conversion to AD dementia.

	HR	95% CI	*p*-Value
Sex (M)	0.52	0.30–0.91	0.02
Age	1.04	1.00–1.08	0.05
MMSE	0.85	0.77–0.94	<0.01
Frailty index	1.11	1.05–1.18	<0.001

*p-Values were obtained from Wald χ^2^ tests, degrees of freedom = 1*.

*CI, confidence interval; HR, hazard ratio; MMSE, Mini–Mental State Examination*.

## Discussion

To our knowledge, this is the first study exploring the impact of the individual’s frailty status and biological decline on the risk of conversion from MCI to dementia. Overall, frailty levels, measured through a FI, resulted to be strongly associated with the risk of cognitive and functional worsening. In fact, subjects with higher FI scores exhibited a significantly increased risk of developing future AD dementia.

Nowadays, special attention is being focused on the possibility of identifying the clinical factors and laboratory findings consenting to early/timely detect those subjects at increased risk of dementia. In this scenario, MCI has increasingly been considered as the optimal phase to explore the clinical and pathophysiological modifications anticipating the onset of overt dementing syndromes (21). To date, most of studies on the conversion of MCI have been concentrated on the contribution of crude socio-demographic (e.g., sex, age, and educational level) and clinical (e.g., comorbidities, neuropsychiatric symptoms) variables, mostly exploring the predictive value of findings and measures individually referring to a specific individual’s health domain (e.g., neuropsychological functions, functional abilities, neuroimaging abnormalities, genetic traits) (22). Nevertheless, existing models of prediction of MCI progression have been shown to have several limitations, including poor discrimination and low positive predictive values (22). Accordingly, the adoption of novel approaches, more properly accounting for the clinical and biological heterogeneity of older people at risk for cognitive decline, has repeatedly been solicited (23).

In this context, the introduction of constructs more broadly reflecting the individual’s frailty status and his/her biological aging may open promising scenarios in the field. This approach may facilitate to multidimensionally capture the pathophysiological complexity of cognitive disorders and neurodegenerative conditions. Moreover, it may consent to more holistically consider the overall health status of the aging individual experiencing the onset of cognitive disturbances, thus not neglecting the multiple and variegate aspects (from sleep disorders to depression, from nutritional deficiencies to polypharmacy) potentially contributing to their occurrence and influencing their phenotypic expression (24). As a proof, in our study, beside the well-established impact of age and baseline cognitive functioning (i.e., MMSE scores), the accumulation of clinical/biological deficits (captured by the FI) significantly influenced the risk of AD dementia. Specifically, FI scores influenced the overall risk of MCI conversion more than age, a well-established risk factor for cognitive decline and dementia. It is noteworthy that the discriminative capacity of the FI was observed despite the cohort being composed exclusively by robust subjects, and was confirmed also among those participants exhibiting the best levels of cognitive performance. These findings are in line with that obtained in cohorts of patients already exhibiting dementing conditions, with frailty measures predicting cognitive outcomes and trajectories (8).

More in particular, our results confirm that the FI may provide useful information when approaching individuals with cognitive disturbances. This model is also easy-to-adopt, being potentially applicable (even retrospectively) from existing datasets and available clinical information. Its use will be even more simplified by the increasing use of electronic medical records (25). In parallel, it can be directly implemented in the clinical practice without requiring changes in the routine/standard approach, not requiring the adoption of specific tests, tools, and *ad hoc* questionnaires, potentially resulting in costly and time-consuming procedures (24).

The present study has some limitations worth to be mentioned. In particular, the small sample size does not consent to draw firm conclusions on the topic. The study population was composed by highly selected MCI subjects attending a university memory clinic, thus with potentially issues in terms of external validity. Moreover, we only focused on the conversion of aMCI to AD dementia, thus not considering the outcomes of different MCI subtypes and the progression toward different dementing conditions.

In conclusions, frailty may significantly increase the individual risk of conversion from a condition of MCI to overt AD dementia. The adoption of constructs comprehensively reflecting the biological decline of the aging subject may add useful estimates and information to those provided by monodimensional variables and traditional cognitive evaluations. In this context, models of frailty (such as the FI) may be easily and promisingly introduced in the neurological practice with the aim of improving both clinical and research standards.

## Ethics Statement

All the data used in the analyses were exclusively retrieved from medical charts where information was recorded as part of the standard clinical routine. The patients and their caregivers (or legal guardians when necessary) provided written informed consent for allowing the utilization of the collected data for research purposes in accordance with the Declaration of Helsinki. The local Ethics Committee, “Comitato Etico Sapienza,” approved the protocol.

## Author Contributions

AT and MC conceived and designed the work, performed the literature search, and wrote the manuscript. LI, LP, and LR collected the neuropsychological data. FD, AC, and VC collected the clinical data. GB and CL participated to the critical appraisal of the available evidence on the topic.

## Conflict of Interest Statement

This research was conducted in the absence of any commercial or financial relationships that could be construed as a potential conflict of interest.
